# Quality Properties of Chicken Emulsion-Type Sausages Formulated with Chicken Fatty Byproducts

**DOI:** 10.3390/foods9040507

**Published:** 2020-04-17

**Authors:** Lina María Peña-Saldarriaga, José Angel Pérez-Alvarez, Juana Fernández-López

**Affiliations:** 1 Research & Development Department, Bios Group, Cra 48 No.274 Sur-89 Envigado, Antioquia, Colombia; lina.pena@grupobios.co; 2 IPOA Research Group, Agro-Food Technology Department, Escuela Politécnica Superior de Orihuela, Universidad Miguel Hernández de Elche, Orihuela, 03312 Alicante, Spain; ja.perez@umh.es

**Keywords:** fatty byproducts, chicken sausages, chicken skin, quality properties

## Abstract

During poultry slaughtering, fatty byproducts are generated, mainly comprising abdominal and gizzard fat, which are mostly discarded and result in consequent environmental problems. The objective of this work was to use these fatty byproducts as fatty raw material in the production of chicken sausages (emulsion-type). They were applied for the partial replacement (40% and 50%) of chicken skin (fatty source usually used in chicken sausages). The effect of these partial replacements on the quality properties (proximate composition, lipid profile, color, texture, and microbiological and sensory properties) of chicken sausages were assessed. Sausages with fatty byproducts added (40% and 50%) showed lower moisture but higher fat content than control. Nevertheless, all of them meet the nutritional requirements of the Colombian regulation for this type of meat product. Sausages with 40% and 50% substitution level showed similar texture properties and microbiological quality as control. When these fatty byproducts were used at 50% substitution level, differences in several color properties and sensorial attributes (color, flavor, and meat taste) were noticed with respect to control. When the substitution level was reduced to 40%, no sensorial differences were detected. Chicken fatty byproducts can be successfully applied as partial replacement of chicken skin in emulsion-type sausages.

## 1. Introduction

Latin American poultry farming will grow at a pace higher than the world average over the next ten years. In this period, global poultry farms will grow 2.5% per year, while in Latin America, the estimate is 4% per year. This major advance in the region is marked by the current economic scenario, in which birds benefit from their greater price competitiveness and consumer preference. In the Latin American market, about 50% of total animal protein consumption is chicken meat. South American countries like Peru, Colombia, and Bolivia have shown significant growth in recent years [1]. In Colombia, poultry farming has been consolidated as a determining factor in the growth of the gross domestic product (GDP) of the agricultural sector [2]. Due to this increase in poultry production, many food industries have focused their strategy on innovation and the development of new poultry meat products [3]. Sausages are one of the oldest forms of meat processing, made by minced meat, salt, spices, and other seasonings stuffed in intestine or artificial case [4,5]. Many varieties have been developed, influenced by climate, religion, availability of ingredients, and processing and preservation methods. Now, it can be said that nearly every culture has its own version of a sausage [4]. It is currently the most produced meat product due the low costs and lack of requirement for sophisticated technology [6]. In addition, it has had a significant increase in consumption throughout the world due to its convenience and practicality [5]. On this point, the market for poultry sausages has been growing not only due to their healthier properties related to meat and fat composition (mainly regarding fatty acids), but also because their consumption is not forbidden by any religion.

An emulsion-type sausage is a mixture of meat, fat, water, spices, and additives in which fat is dispersed more or less uniformly in a continuous, highly hydrated protein matrix. The fat droplets do not necessarily remain globular, and they may coalesce with each other, but they cannot escape from the matrix to produce a single phase. The desirable properties of emulsion-type sausages are largely determined by the stability of moisture and fat binding in the highly hydrated gellable protein matrix. In addition, the moderate denaturalization of muscle proteins during the thermal process gives a fine texture and flavor to emulsion-type sausages. In this type of sausage, fat is an essential component because it improves the tenderness, juiciness, and overall palatability [7,8,9]. Traditionally, in most chicken sausages, the used source of fat does not come from chicken but mostly from pork (back fat) or, in some cases, beef (tallow) [10,11,12] mainly due to their superior technological characteristics [8,9] and, also, their greater availability. Nevertheless, the nutritional quality of these fatty sources (regarding their high fatty acid composition) and, also, some religion demands are the main inconvenience nowadays. For this reason, chicken skin is being used as the main fatty source in chicken sausages.

On the other hand, some of the byproducts generated during poultry slaughtering (viscera, bones, head, cartilage, crest, blood, abdominal fat, feet, and fathers), which represent almost 37% of the total live weight of the animal [13], could be reused, increasing their nutritional and environmental value and providing sustainable development for food industries and supporting the value chain in this sector [14]. More specifically, the abdominal and gizzard fat that remain inside the poultry carcass could be used as a fatty source for the production of chicken sausages or other meat products, mainly considering their characteristic content in monounsaturated fatty acids and vitamin A [15]. Until now, this abdominal and gizzard fat has been discarded by small producers together with viscera, feathers, and blood, creating an environmental problem, or in some cases, are sold to outlets such as animal feed and pet food processors [16,17]. The aim of this work is to evaluate the effect of the partial substitution of chicken skin by fatty byproducts (abdominal and gizzard fat) on the quality of a traditional Colombian sausage (emulsion-type).

## 2. Materials and Methods

### 2.1. Sausages Preparation

Chicken sausages were prepared in a production plant (Frico, Medellin, Colombia) following industrial production practices. The formulations of the chicken sausages are shown in Table 1. As can be seen in this table, the sausages were made from chicken breast (35.8%), mechanically deboned chicken meat (15%), and chicken fat (22.5%). Three treatments were conducted depending on the chicken fat source: control (chicken skin as fat source), 40SFB (40% of chicken skin was substituted by chicken fatty byproducts), and 50SFB (50% of chicken skin was substituted by chicken fatty byproducts). The chicken breast and skin were ground in a JIMO JR 12 grinder (Torrey, México) with a mesh diameter of 5 mm previously to be added to the cutter (Cruells, Girona, Spain). All ingredients were added to the cutter with 120 kg capacity, following the order and mixing times indicated in Table 1. The temperature of the emulsion was controlled and maintained below 10 °C by monitoring with a digital temperature probe during the process. The meat emulsion is formed when the proteins are solubilized and the fat particles suspended and entrapped within the protein matrix. Then, the meat batter was stuffed into artificial casings (45 mm diameter, ALIFLEX coextruction casings in polyamide and polyolefins, Alico S.A., Medellín, Colombia) using a hydraulic stuffer (AK Ramón, Barcelona, Spain) and manually clipped pieces of approx. 500 g, and cooked in an oven (CI Talsa, Medellín, Colombia) until reaching 75 °C at the center of the sausage (approx. 30 min). After cooking, sausages were immediately cooled in an ice bath and stored at 4 °C until analysis. Processing was repeated three times with each formulation.

### 2.2. Proximate Composition

The proximate composition of chicken sausages was determined following the methodologies described by AOAC [18] for moisture content (No. 950.46.41), ashes (No. 920.153), proteins (No. 928.08), and fats (No. 991.36).

### 2.3. Color Evaluation

For color measurements, sausages were cut to approx. 2.5 cm thickness and the measurements were made directly on the internal part of the sausage using a spectrocolorimeter SP62 (X-RITE, Grand Rapids, MI, USA) with a 64 mm diameter illumination area. Color measurements were based on the CIELAB color space, selecting illuminant D_65_ and 10° observer, following American Meat Science recommendations [19]. The following color coordinates were determined: lightness (L*), redness (a*, ± red-green), and yellowness (b*, ± yellow-blue). The chroma saturation index (C* = (a*^2^ + b*^2^)^1/2^), the hue angle (h* = tan^−1^ b*/a*), and the color differences (ΔE* = (ΔL*^2^ + Δa*^2^ + Δb*^2^)^1/2^) were also estimated with respect to control sausages.

### 2.4. Texture Profile Analysis

The texture profile analysis (TPA) was selected for evaluating sausage textural properties [20]. Samples were cut into 25 mm cubic sections (height × length × width). The textural properties for each sample were measured using a cylinder probe (35 mm diameter) set attached to a Texture Analyzer (TA-XT2i, Stable Micro System Ltd., Surrey, UK). The following test conditions were applied: stroke, 1.5 kg; test speed, 4 mm/s; distance, 10 mm (40% compression). Although TPA gives the values of 7 parameters, the most commonly discussed in reference to meat protein gelation are the following: hardness [peak force on first compression (N)], cohesiveness [ratio of active work done under the second force–displacement curve to that done under the first compression curve], springiness [ratio of the sample recovered after the first compression], gumminess [hardness × cohesiveness], and chewiness [hardness × cohesiveness × springiness (N)] were computed.

### 2.5. Microbiological Analysis

A 25 g sample of each product was taken aseptically by scalpel excision and placed in a sterile Stomacher bag containing 225 mL of peptone water (PW, Oxoid Ltd., Hampshire, England). The samples and the PW were stomached (Stomacher 400, A.J. Seward, London, England) for 2 min. Decimal dilutions were carried out using the same diluent. All dehydrated microbiological media, except for Sulfite Polymyxin Sulfadiazine Agar (SPS agar) and chromID^®^ Coli (Coli ID) were from Oxoid (Basingstoke, UK), whereas SPS agar was from Merck (Darmstadt, Germany) and Coli ID from Biomeriux (Lyon, France). Mesophiles were determined using plate count agar spread plates incubated at 30 °C for 72 h (mesophiles) [21]. *Salmonella* was cultured using Brilliance™ Salmonella Agar for 24 h at 37 °C [22]. *Listeria monocytogenes* was cultured using Listeria Oxford Agar for 48 h at 30 °C [23]. Spores of sulfite-reducing *Clostridium* were cultures with SPS agar incubated at 35 °C in an anaerobic atmosphere for 72 h [24]. Total coliforms and *Escherichia. coli* were cultured using Coli ID chromogenic medium incubated at 37 °C for 22 h [25]. Baird Parker Agar was used for the detection and enumeration of coagulase-positive staphylococci (incubated at 35 °C for 48 h) [26]; positive strains were confirmed by the coagulase test. All assays were performed according to the Bacteriological Analytical Manual of the U.S. Food and Drug Administration [27]. Microbial counts are expressed as CFU/g.

### 2.6. Sensory Evaluation

For the sensorial analysis of sausages, descriptive and quantitative tests were applied following ISO 4121 [28]. Sensory analyses were conducted by 7 trained panelists from the Sensorial Analysis Laboratory at the Food Science and Technology Institute (INTAL, Colombia). Each sausage was warmed (17 ± 2 °C), sliced into 10 mm thick slices (25 g approx.), coded randomly, and served to panelists. The following descriptors were selected for the sausage evaluation: color, flavor, typical meat taste, saltiness, off-taste, condiment taste, hardness, chewiness, and fat mouthfeel. A 7-point scale was used to evaluate the attributes with the following degrees of intensity: 1 = none, 2 = slight, 3 = slight-medium, 4 = medium, 5 = medium-high, 6 = high and 7 = intense. At the end, each panelist was asked about the overall quality of the product (low, medium, or high). Panelists cleansed their palate with water between samples.

### 2.7. Statistical Analysis

One-way analysis of variance was carried out to verify the effect of sausage formulation on chemical, physicochemical, microbiological, and sensory properties. Three samples, each contained three replicates for the experiment, were analyzed in triplicate. Tukey’s test was performed when ANOVA revealed significant differences (*p* < 0.05) between treatments. The SPSS software (v. 26.0) was used to perform the statistical test.

## 3. Results & Discussion

### 3.1. Proximate Composition

Table 2 shows the proximate composition of chicken sausages as affected by the fat source used. Sausages with fatty byproducts added (40SFB and 50SFB) showed lower moisture content but higher fat content than control (*p* < 0.05), while the protein and ash contents were not affected (*p* > 0.05) by this substitution. These differences are related to the different composition of skin and fatty byproducts. Skin has higher moisture content but lower fat content than fatty byproducts [29,30]. Chicken sausages with 40% and 50% substitution levels (40SFB and 50SFB) only showed differences (*p* < 0.05) in fat content: the higher the substitution level, the higher the fat content. This trend regarding the behavior of fat and moisture in meat products depending on the percentages of added fats agrees with the reports of other authors for several meat products [31,32]. For this reason, nutritional requirements for cooked meat products in several official regulations are expressed as minimal protein content, maximal fat content, and maximal fat + moisture content. In this case, all sausages showed values within the ranges allowed by the Colombian regulation for cooked sausages (protein > 10%, fat < 28%, fat + moisture < 90%) [33]. Taking into account that the lipid profile of chicken fat has been reported as highly healthy, mainly due to the predominance of unsaturated fatty acids (65.5%) over saturated fatty acids (30.3%) [30,34] without significant differences from different chicken carcass parts (skin, adipose tissue, and meat) [29,30,35], no differences in the lipid profile of these sausages would be expected.

### 3.2. Color Properties

Color is regarded, first, as a qualitative criterion in meat products and plays a key role in consumer’s perception and product acceptability [36]. Color parameters of sausages are shown in Table 3. Saturation index (C*) was the only color parameter that was not affected (*p* > 0.05) by the substitution of chicken skin by fat byproducts at any of the studied concentrations. The highest differences in the color parameters (respect to control) were observed in sausages with 50% substitution. These sausages were lighter and showed less redness and high yellowness and hue than control (*p* < 0.05). On the contrary, when fatty byproducts were added at 40% substitution, only yellowness and hue values were significantly affected (*p* < 0.05). Modifications in lightness and a* and b* coordinates in meat products have been related to the fat content; the higher the amount of fat, the lower redness but the higher the L* and b* values. According to Hughes et al. [37], it was noted that reducing the fat content resulted in a decrease in both lightness and yellowness as well as an increase in the redness values of Frankfurt sausages. This pattern has been reported in several comminuted and emulsified meat products by other authors [6,38,39]. Chicken skin has less than 30% fat content in comparison with 75% in fatty byproducts [29,30]. These differences between the yield for lipid extraction between chicken skin and fat byproducts could be responsible for these color modifications in the sausages.

**Figure 1:**
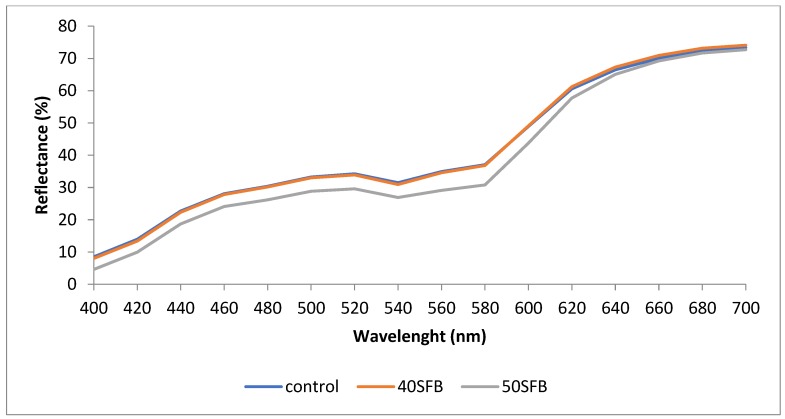
Reflectance spectra (360–740 nm) of chicken sausages [control, 40SFB (40% substitution of chicken skin by fatty byproducts and 50SFB (50% substitution of chicken skin by fatty byproducts)].

Looking the reflectance spectra (Figure 1) of the sausages, it can be also observed that sausages with 50% substitution showed lower (*p* < 0.05) percentages of reflectance, for all wavelengths, than the control and 40% substitution sausages (without significant differences between them). In spite of these changes, it could be said that the three spectra kept the typical shape attributed to the meat reflectance spectrum without being affecting by the level of chicken skin substitution. It must be taken into account that this typical shape of the meat reflectance spectrum is determined by the major pigment in meat (myoglobin) and by the relative amounts of its three forms, i.e., deoxymyoglobin, metmyoglobin, and oxymyoglobin and their interconversions and degradations through oxygenation, oxidation, and reduction reactions, ultimately influencing the appearance of meat color [19].

### 3.3. Texture Properties

Texture properties of sausages (hardness, cohesiveness, gumminess, springiness and chewiness) were not affected (*p* > 0.05) by the replacement of chicken skin by fatty byproducts at any level. The following overall values of TPA parameters obtained for chicken sausages were hardness: 497.4 ± 92.9 N; springiness: 0.75 ± 0.11 mm; cohesiveness: 0.18 ± 0.09; gumminess: 89.5 ± 9.7 N; and chewiness: 53.9 ± 6.50 N mm. Several modifications in texture properties have been reported in cooked meat products regarding formulation modifications in reference not only to the type of ingredient but also to its concentration [6,39,40,41,42]. Texture in comminuted and cooked meat products has been related to the ability of meat proteins to bind to water and fat and create a protein matrix in addition to their stability during processing (addition of salt, non-meat binders, heat treatment, etc.) affecting their emulsifying and gelling properties in the meat batter. Water and fat can interact with other ingredients to develop a desirable texture and mouthfeel and vitally influence the overall quality of the product. In this case, the partial replacement of chicken skin by chicken fat byproducts did not modify the sausage’s texture, which could be attributed to the high stability of the protein matrix, which is able to bind water and fat regardless of the fat source used. These results could indicate the feasibility of chicken skin replacement (up to 50%) by fat byproducts in sausage.

### 3.4. Microbiological Analysis

The substitution of chicken skin with chicken fat byproducts (at any level) did not influence (*p* > 0.05) the microbiological quality of chicken sausages. Table 4 shows the microbial counts of chicken sausages. All these values were within acceptability limits prescribed by Colombian legislation [33].

### 3.5. Sensory Evaluation

**Figure 2:**
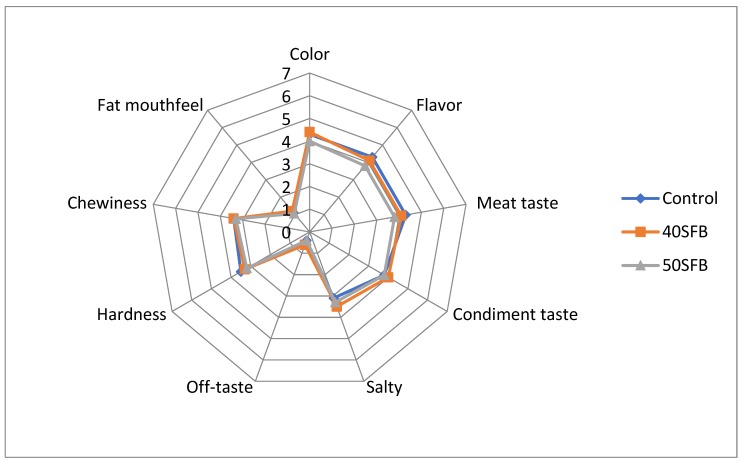
Sensory evaluation of chicken sausages [control, 40SFB (40% substitution of chicken skin by fatty byproducts and 50SFB (50% substitution of chicken skin by fatty byproducts)].

The results of sensory assessment are shown in Figure 2. No significant changes (*p* > 0.05) were observed in the scores of all the evaluated attributes between control and 40% substitution chicken sausages. No significant differences (*p* > 0.05) were found in terms of fat mouthfeel, chewiness, hardness, off-taste, saltiness, and condiment taste amongst all groups. However, 50% substitution sausages showed significant differences (*p* < 0.05) in terms of color, flavor, and meat taste. These 3 attributes were scored worse by the panelists for the 50% substitution sausages. The results obtained for the sensorial evaluation of texture (chewiness and hardness) are in agreement with the instrumental texture measure because texture differences between chicken sausages were not detected in any case.

The objective color measure of sausages is also in agreement with sensorial results. Although in 40SFB sausages, the objective color measure showed significant differences with control samples for yellowness and hue values, their color differences (ΔE*) with respect to control were lower than 3 units (Table 3), which is under the level that could be detected by human eye and, so, these differences could not be detected by sensorial analysis. On the contrary, 50SFB sausages showed higher color differences (3.3; Table 3) contributing to being perceived as different by panelists.

## 4. Conclusions

The results of this study show that chicken fatty byproducts could be used in place of chicken skin in chicken emulsion-type sausages. Sausages with fatty byproducts added (40SFB and 50SFB) showed lower moisture but higher fat content than control. Nevertheless, all of them meet the nutritional requirements of the Colombian regulation for this type of meat products. When these fatty byproducts were used at 50% substitution level, several differences in color properties (but without affecting the typical shape of reflectance spectrum in this type of meat product) were noticed with respect to control, and differences in color, flavor, and meat taste were also detected by panelists. Therefore, when the substitution level was reduced to 40%, no sensorial differences were detected with respect to control sausages. This study suggests that chicken sausages with 40% substitution of chicken skin by chicken fatty byproducts is a highly marketable option.

## Figures and Tables

**Table 1:** Formulation of chicken sausages (40SFB: 40% substitution of chicken skin by fatty byproducts; 50SFB: 50% substitution of chicken skin by fatty byproducts).

Ingredient	Mixing Time (min)	Control	40SFB	50SFB
		kg	kg	kg
Chicken breast	10 min	43	43	43
MDM ^1^(chicken)		18	18	18
Sodium lactate		2.3	2.3	2.3
Water		3	3	3
Salt		0.25	0.25	0.25
Cured salt (12% potassium nitrate)		0.2	0.2	0.2
Accord phosphates		0.54	0.54	0.54
Flavor mix for sausages ^2^		1.51	1.51	1.51
Isolate soya protein	2 min	2.5	2.5	2.5
Binder XT 202		4.8	4.8	4.8
Water	4 min	4	4	4
Acid violet 051 (added to water for dilution)		30 mL	30 mL	30 mL
Water	4 min	4	4	4
Wheat flour		4.5	4.5	4.5
Sodium ascorbate		0.40	0.40	0.40
Wheat starch		4	4	4
**Chicken skin (minced)**		27	16.2	13.5
**Chicken fat byproducts**			10.8	13.5
**TOTAL**	20 min	120	120	120

^1^ MDM: mechanically deboned meat. ^2.^ Flavor mix for sausages: coriander, mace, cumin, BHT, citric acid, silicon dioxide and liquid smoke.

**Table 2:** Proximate composition of chicken sausages with different substitution level (%) of chicken skin by chicken fatty byproducts (40SFB: 40% substitution of chicken skin by fatty byproducts; 50SFB: 50% substitution of chicken skin by fatty byproducts).

	Moisture (%)	Fat (%)	Protein (%)	Ash (%)
**Control**	62.17a	10.14c	13.04a	3.42a
**40SFB**	61.63b	13.08b	12.54a	3.52a
**50SFB**	61.40b	14.67a	12.43a	3.42a

a–c: Different letters in the same column indicate significant differences (*p* < 0.05).

**Table 3:** Color parameters of chicken sausages with different substitution level (%) of chicken skin by chicken fatty byproducts (40SFB: 40% substitution of chicken skin by fatty byproducts; 50SFB: 50% substitution of chicken skin by fatty byproducts).

SAMPLE	L*	a*	b*	C*	h*	ΔE*
CONTROL	66.41 ± 0.21b	13.33 ± 0.09a	18.76 ± 0.15b	23.00 ± 0.15a	54.60 ± 0.20b	
40SFB	67.62 ± 0.25b	13.42 ± 0.11a	19.70 ± 0.20a	23.82 ± 0.23a	56.73 ± 0.25a	2.2
50SFB	69.42 ± 0.26a	12.51 ± 0.12b	19.93 ± 0.17a	23.53 ± 0.19a	57.87 ± 0.15a	3.3

a–b: Different letters in the same column indicate significant differences (*p* < 0.05).

**Table 4:** Microbial counts in chicken sausages.

Microorganisms	Results (CFU/g)		
	CONTROL	40SFB	50SFB
Mesophiles	1234 ± 130	1086 ± 180	1160 ± 147
Total coliforms	<10	<10	<10
*E. coli*	<10	<10	<10
Coagulase-positive *Staphylococcus*	<100	<100	<100
*Salmonella*	Absence/25 g	Absence/25 g	Absence/25 g
*Listeria monocytogenes*	Absence/25 g	Absence/25 g	Absence/25 g
Spores of sulfite-reducing *Clostridium*	<10	<10	<10
